# P300 as a neural indicator for setting levels of goal scores in educational gamification applications from the perspective of intrinsic motivation: An ERP study

**DOI:** 10.3389/fnrgo.2022.948080

**Published:** 2022-10-21

**Authors:** Hiroki Watanabe, Yasushi Naruse

**Affiliations:** Center for Information and Neural Networks, Advanced ICT Research Institute, National Institute of Information and Communications Technology, Osaka University, Kobe, Japan

**Keywords:** P300, feedback-related negativity (FRN), intrinsic motivation, achievement goal, gamification, event-related potentials (ERPs), electroencephalogram (EEG), self-determination theory (SDT)

## Abstract

The challenge level of goal achievement affects intrinsic motivation. Thus, the goal score learners are required to achieve is an important element in gamified educational applications to foster users' intrinsic motivation. However, determining optimal goal scores that enhance the intrinsic motivation of each learner is not easy because individual competence and preferences for the challenge level (e.g., preference for difficult-to-achieve challenges) vary. One approach is to determine the goal score using physiological measurements to estimate when an individual's intrinsic motivation is reinforced. Measurement of event-related potentials (ERPs) is considered useful for this purpose. ERPs time-locked to feedback onset, such as feedback-related negativity and P300, reflect intrinsic motivation. However, it remains unclear whether these ERPs can serve as indicators of optimal goal scores for gamified educational applications in terms of intrinsic motivation. The present study aimed to examine whether ERP measures vary with the challenge levels of the goal score determined by participants' competence (too-easy, moderate and too-hard levels) and/or with their preference for these levels when using a gamified mental arithmetic application. Thirty-three participants solved 64 addition problems in one session in this application and received auditory feedback immediately after each answer entry. Scores were then calculated based on their task performance. Before each session, participants were informed of the goal score and instructed to exceed it as much as possible. Sessions were repeated six times at easy, moderate, and hard levels of goal scores, with two sessions per level. Goal score preferences were quantified based on subjective ratings of the motivation to achieve each level of goal score using a 7-point Likert scale. The mean amplitudes of ERPs were obtained for each participant. Results showed that P300 was significantly related to subjective ratings but not to levels of goal scores, indicating that P300 could be an indicator of participant preference for goal score levels. This study suggests that measurement of P300 may serve as a neural indicator providing an optimal goal score for individual learners that maximizes their intrinsic motivation in gamified learning applications.

## 1. Introduction

The challenge level of activities influences people's intrinsic motivation—spontaneous engagement in an activity for fun, satisfaction, and challenge without expecting any other outcome (Ryan and Deci, 2000a). Self-determination theory (SDT) (Deci and Ryan, 1985; Ryan and Deci, 2000b) suggests that fulfilling the psychological need of competence is important for human motivation, along with relatedness and autonomy. Activities at the optimal challenge level—not too easy and not too hard—are associated with the satisfaction of competence (Deci and Ryan, 1985; Ryan and Deci, 2000b). Thus, setting the optimal challenge level is expected to reinforce intrinsic motivation. However, establishing the appropriate level is not easy because the competence in an activity depends on the individual, and there may be differences in the level of difficulty perceived as optimal. At the same time, the relationship between intrinsic motivation and challenge level suggests that the optimal level of challenge may be estimated by noting the degree of intrinsic motivation when individuals engage in activities at different levels. For example, Rani et al. (2005) adjusted the difficulty levels of a computer game based on users' anxiety levels estimated from physiological measurements from electrocardiograms or electromyograms, and so on. The adjustments resulted in improvements in the game performance and greater perceived challenge. Given the real-world applications of such a system, physiological data may have advantages over reliance on subjective evaluation because measuring physiological responses during a task allows for online estimation without imposing additional tasks and with less subjective bias.

Recent studies have shown that event-related potentials (ERPs), such as feedback-related negativity (FRN) and P300, can serve as physiological indicators of intrinsic motivation. FRN is observed in the fronto-central region at approximately 250 ms from the onset of the negative feedback presentation (Miltner et al., 1997; Gehring and Willoughby, 2002). Holroyd and Coles (2002) explained this ERP component in terms of reinforcement learning elicited when the outcome of an event is worse than predicted. Consistent with reinforcement learning, FRN reflects extraction of motivationally significant outcomes (Pfabigan et al., 2010). Recent research has demonstrated that FRN amplitudes calculated from the difference wave between the ERP to incorrect feedback and one to correct feedback are related to intrinsic motivation (Ma et al., 2014a; Fang et al., 2018; Meng et al., 2021). The FRN response is also modulated by affective mood states, such as positive emotion (Zhao et al., 2016; Paul and Pourtois, 2017) or boredom (Milyavskaya et al., 2019), which are presumably induced by the degree of intrinsic motivation. P300 is observed in the centro-parietal region approximately 300 ms after stimulus onset and reflects the allocation of attentional resources to the stimulus (Nieuwenhuis et al., 2005; Polich, 2007). P300 is expected to serve as an indicator of intrinsic motivation because heightened motivation induces greater attention to outcome feedback (San Martín, 2012). In addition, P300 is also sensitive to the motivational significance of the stimuli (Nieuwenhuis et al., 2005). Indeed, Fang et al. (2020) demonstrated that the decrease in human motivation due to autonomy frustration led to a decrease in the amplitude of P300 time-locked to feedback onset in the subsequent task. P300 amplitude was also modulated by the affective significance of the outcome, enhanced by changing the initial choice in the gambling task (Zhou et al., 2010) and incidental negative emotion (Zhao et al., 2016). In addition to FRN and P300, the N1 component of the stimulus onset observed at approximately 100 ms, reflects the top-down attention to the stimuli (Hillyard et al., 1973) and possibly reflects intrinsic motivation.

If ERP measurements can serve as indicators of intrinsic motivation, they could be used for more effective learning in educational settings. In recent years, the concept of gamification—“the use of game design elements in non-game contexts” (Deterding et al., 2011) to promote intrinsic motivation by enhancing task enjoyment—has become widespread and its effectiveness has been investigated in the fields of education and learning (Hamari et al., 2014). The challenge level is also important in gamified learning content because Hamari et al. (2016) showed that perceived challenge affected engagement, immersion, and perceived learning in gamified learning using video games. Therefore, establishing a physiological measure of intrinsic motivation may lead to appropriate challenge levels for individuals engaged in gamified learning content, increasing their intrinsic motivation for the task.

However, it remains unclear whether variation in intrinsic motivation—as a function of the challenge level of the goal scores to be achieved by the user in a gamified educational application—is reflected in neural indicators. Previous ERP research on intrinsic motivation focused on the effects of autonomy frustration (Fang et al., 2020), competence frustration (Fang et al., 2018, 2021; Meng et al., 2021), or monetary reward (Ma et al., 2014a) on intrinsic motivation. Meng et al. (2016) defined challenge level as the differences in task competence compared with an opponent in a stopwatch game. Results demonstrated that the challenge level modulates intrinsic motivation (Meng et al., 2016). However, the challenge levels of goal scores set to be exceeded by the user in gamification have not yet been the subject of ERP studies. Goal-setting theory (Locke and Latham, 2002) states that setting concrete goals is more motivating than abstract goals such as “doing your best," and this principle also applies to gamification (Tondello et al., 2018). Setting concrete goal scores for users is considered an important element in gamified educational applications to foster users' intrinsic motivation.

To determine goal scores in gamified educational applications, the SDT predicts that a moderate goal score (i.e., not too easy or too hard for the user to achieve) promotes intrinsic motivation. However, while setting a moderate goal score is generally considered motivating, the possibility of individual differences in preferences for the level of goal scores cannot be excluded. Some people prefer goal scores that are very hard to achieve, while others prefer more easily achievable levels. If such preferences for the level of goal scores can be postulated, the neural indicators of intrinsic motivation would be expected to be related to the preference. That is, we would expect a correlation of ERP with the subjectively rated preferences for too-easy, moderate, or too-hard goals rather than the challenge level of the goal scores being determined by the individuals' competence (i.e., differences in the ERP across the too-easy, moderate, and too-hard goals). If neural indicators vary with the individual preference for the levels of goal scores in the gamified educational applications, as shown by Rani et al. (2005), an optimal goal score for an individual learner may be provided in applications by enhancing intrinsic motivation. However, to our knowledge, the ability of these neural indicators to reflect the challenge level of the goal score and/or individual preference has seldom been directly investigated.

The effectiveness of these ERP indicators has also not been evaluated in real-world settings where gamified educational applications are used. Most previous research has focused on the stopwatch game task, adopted by Murayama et al. (2010) requiring participants to stop a stopwatch precisely at a specified time. With advances in EEG measurement systems, recent research, however, has proven the applicability of EEG-based state estimation in situations that mimic a real-world environment (Watanabe et al., 2021a,b). In addition, recent studies have shown that the FRN is observable in gaming tasks using virtual reality (Yokota and Naruse, 2021). Nonetheless, whether these neural indicators work effectively in actual gamification educational applications remains unclear.

Based on the knowledge gaps described, we examined whether ERP neural indicators (1) vary based on the challenge level of the goal scores determined by participants' competence, and (2) are related to the preference for goal score levels in a gamified educational application. For this purpose, we prepared a graphical user interface (GUI) application that gamified the “Hundred-Square Calculations" mental arithmetic task devised in Japan to cultivate the foundations of calculation skills. In this task, participants repeatedly solve mental arithmetic (addition) problems in a single session and receive auditory feedback immediately after entering the answer to a question. Scores earned were calculated based on performance in a single task session. Three levels of goal scores were prepared (easy, moderate, and hard goal scores to achieve) based on participants' competence estimated from the practice session. Participants performed a series of task sessions at those three levels of goal scores, during which electroencephalogram (EEG) measurements were taken. Preference for the goal score levels was quantified using the subjective rating data on achievement motivation for each level of goal score. We analyzed whether there is a relationship between the level of goal score and/or subjective ratings with these ERP neural indicators.

## 2. Methods

### 2.1. Participants

Thirty-three healthy adults participated in the current study (16 females). Their mean age was 26.7 years old [standard deviation (SD) = 6.86], and the age range was from 20 to 39 years old. They had normal or corrected-to-normal vision and reported no medical history of neurological disorders. Recruitment took place by accessing individuals registered with a staffing agency. All participants provided written informed consent to participate in the study. The study was approved by the Ethics Committee for Human and Animal Research of the National Institute of Information and Communications Technology.

### 2.2. Data acquisition

Considering the usability of ERP indicators in a real-world environment, EEG data were measured from a small number of electrodes, as in our previous studies (Ihara et al., 2021; Watanabe et al., 2021a,b; Yokota and Naruse, 2021). We used three active dry electrodes placed at Fz, Cz, and Pz according to the international 10–20 system, and a wireless portable system (PolymateMini AP108, Miyuki Giken Co., Ltd.). The locations of the three electrodes were selected because N1/FRN and P300 are distributed in the fronto-central and parietal regions, respectively. Two electrodes were placed on the outer canthi and above participants' left eyes to monitor horizontal and vertical electrooculograms (EOG). The ground and reference electrodes were placed on the left and right earlobe, respectively. The sampling rate was 500 Hz.

### 2.3. Experimental task

**Figure 1:**
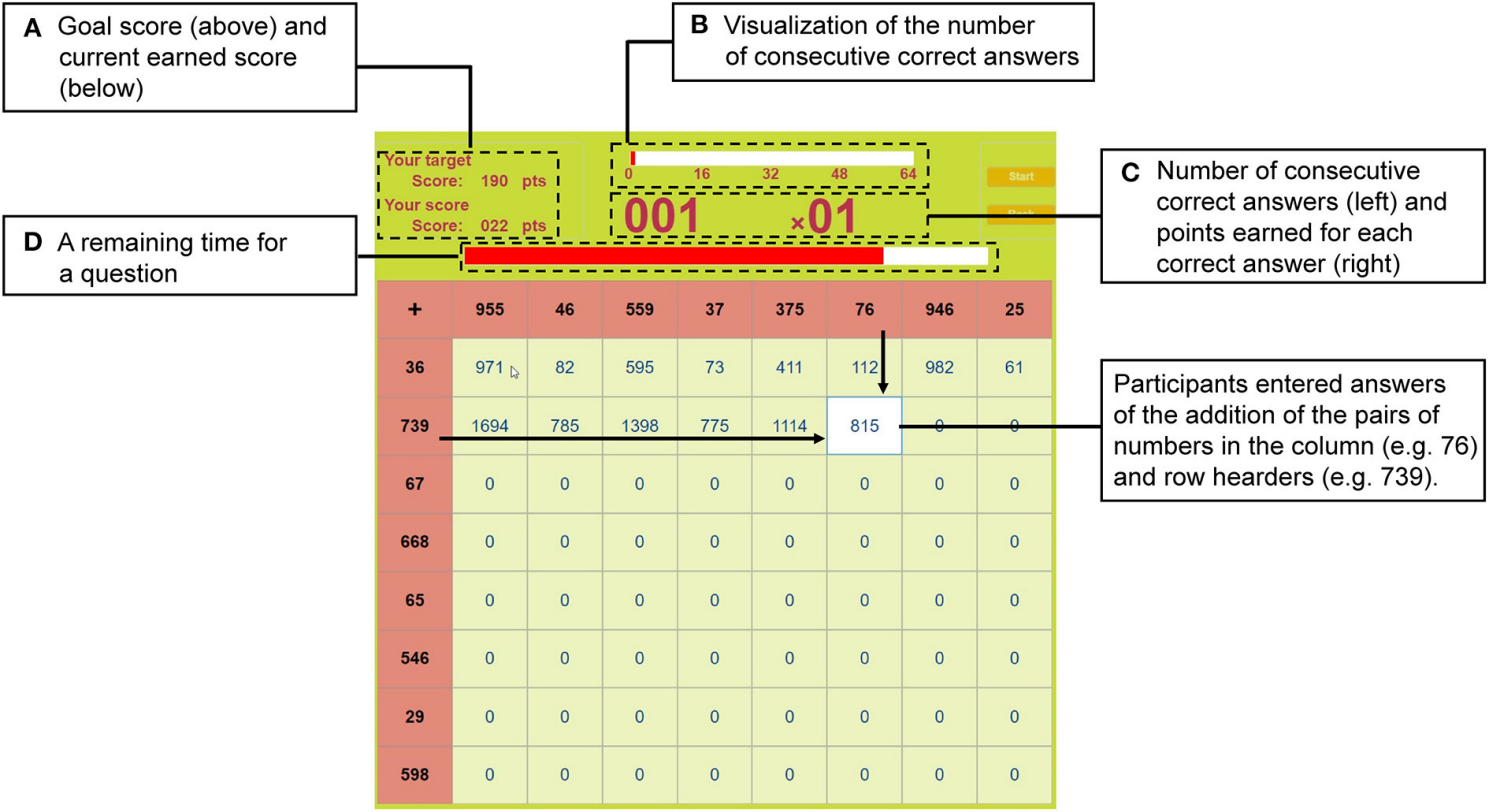
The appearance of a gamified mental arithmetic graphical user interface application. The participant enters answers to addition sums using the number pairs in the column and row headers in sequence. After entering the answer, auditory feedback on the correctness of the answer is immediately provided. The goal score and the current score earned are shown in the upper left area **(A)**. Participants were instructed to confirm the goal score before starting the task and to try to exceed the goal score as much as possible. The number of consecutive correct answers is visualized by a bar **(B)**. Below the bar, the current number of consecutive correct answers and the points earned for each correct answer are displayed **(C)**. The points earned vary depending on the number of consecutively correct answers. The remaining time for each question is displayed as a progress bar **(D)**. If an answer is not entered within the time limit, negative feedback is returned, and the participants are automatically moved to the next question. The dashed squares are shown for illustration purposes only.

The appearance of the gamified mental arithmetic task is shown in Figure 1, with a description of the game elements. The task was implemented as a custom desktop application using MATLAB (The MathWorks, Inc.). Participants were instructed to achieve the goal score displayed on the application (Figure 1A) as best they could and input the results of the addition of every pair of numbers displayed in the row and column headers into the table. The calculation was started by pressing the start button using the mouse and adding the first number in the row header and the numbers in each column header in order from left to right. After adding the numbers in the column headers, the calculation was moved to the next number in the row header. The numbers in the row and column headers were randomly determined per session, participant and set, so that three- and two-digit numbers appeared alternately. As a result, three types of addition problems were presented: pairs of three-digit numbers (16 calculations), pairs of three-digit and two-digit numbers (32 calculations), and pairs of two-digit numbers (16 calculations; c.f. Figure 1). The first digit was set to be five or more so that carry-over from the first digit to the next digit would always occur. This difficulty level of the task was determined based on the preliminary experiment results, ensuring it was neither too difficult nor too easy. There are usually 100 sums in the “Hundred-Square Calculations," but considering the time required for the experiment and participants' concentration, 64 questions were prepared for a session. Participants used a numeric keypad to input their answers. Negative and positive auditory feedback for incorrect and correct answers was given to the participants immediately after they entered their answers by pressing the Enter key. Scores earned were based on the number of correct answers in each session.

Several elements were included to gamify the mental arithmetic task. First, the point earned for each correct answer was increased according to the number of consecutive correct answers. If the number of consecutive correct answers was less than five, participants got one point for each correct answer. For each consecutive correct answer that exceeded a multiple of five, the number of scores increased by a multiple of four. If the number of consecutive correct answers exceeded 50, the point for a correct answer was 40. In other words, if the number of consecutive answers was in the range of [1, 5), [5, 10), …, [50, 64], the point for a correct answer would be 1, 4, …, 40, respectively. The points earned for the correct answer and the number of consecutive correct answers were displayed during the task (Figures 1B,C). Second, a time limit for each question was set to 10 s. If an answer was not input within the time limit, negative feedback was given, and the time for the next question automatically started. The remaining time for a question could be viewed on the progress bar above the table (Figure 1D).

### 2.4. Procedure

**Figure 2:**
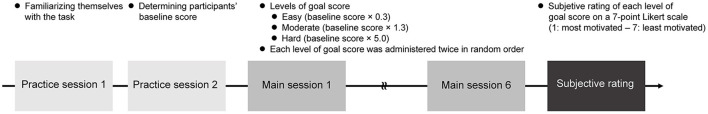
Data collection procedure. Participants performed two practice sessions, one to familiarize themselves with the task and the other to determine the baseline score. In the main sessions, they performed a mental arithmetic task with three levels of goal scores: easy (baseline score × 0.3), moderate (baseline score × 1.3), and hard (baseline score × 5.0). Each level of goal score was administered twice in random order. After completing the task, the participants subjectively rated how motivated they were to achieve the goal score, using a 7-point Likert scale.

Data collection was carried out in a dimly lit and soundproofed room. Participants ran the application for the mental arithmetic task on the monitor display. A within-subjects design was employed for the experimental procedure, with participants performing the task for each level of goal scores. The EEG data collection consisted of two practice sessions and six main sessions, followed by a subjective rating of each level of goal score (Figure 2). In the first practice session, participants familiarized themselves with the GUI application. If they found the task very difficult during the first practice and the percentage of correct answers was low (<10%), the time limit was extended to 12 s. Five participants used the extended time limit. In the second practice session, the participants performed the task the same way as in the main session. The score in this second practice session was used as a baseline to set goals for the main session.

In the main session, the task was conducted under three levels of goal scores (easy, moderate, and hard) aimed at varying participants' intrinsic motivation for the task. The baseline score defined the criterion of each level of goal score: a baseline score × 0.3 for easy, × 1.3 for moderate, and × 5.0 for hard. The goal score was displayed on the application before the session (Figure 1A). Participants were instructed to confirm the score before starting the session and try to achieve or exceed that goal score. Two sessions were conducted for each level of goal score (hereafter referred to as the first and second attempts per level of goal score; six main sessions in total). The order of the level of goal scores was randomly determined for each participant, and every session included a short break. Participants were discouraged from making large body movements and being overly tense during the session to avoid artifacts from manipulating the GUI application. Speaking the numbers out loud or counting on fingers was also prohibited. After completing the mental arithmetic task, participants subjectively rated how motivated they were to achieve the goal score for each level on a 7-point Likert scale (1: most motivated–7: least motivated). The entire data collection procedure lasted approximately 2.5 h, including preparation.

### 2.5. Statistical analysis

First, we determined whether the levels of goal scores modulated participants' subjective intrinsic motivation using the subjective rating data. We used a Friedman test, a non-parametric test, because the subjective rating data were not normally distributed. Next, we aimed to determine whether the levels of goal scores (Goal: easy, moderate, and hard) and/or the preference for these goal levels (i.e., subjective rating data; Rating: a continuous variable) affected behavioral performances and ERP responses. The statistical tests were conducted on each behavioral performance measure, such as scores, the frequency of negative feedback in the task and the mean amplitudes of N1, FRN, and P300. To eliminate the possibility of any confounding differences in performance that would affect the ERP responses, we also investigated whether the behavioral factors potentially influencing ERPs differ by Goal and/or are related to Rating. For these behavioral and ERP analyses, we used a linear mixed-effects model (LME) or generalized linear mixed-effects model (GLME) depending on the data distribution. The factors of Goal and Rating were included as fixed effects. The subjective rating data were centered so that the mean was zero when it was used as a fixed effect. A random intercept was specified for each participant in the (G)LMEs. We used the conditional Akaike information criterion (cAIC) (Vaida and Blanchard, 2005) to determine whether a random slope for Rating should be included. The Type III *F*-test using the Kenward-Roger method and the Type III likelihood ratio test (LRT) were used for determining the significance of the fixed effects in the LME and GLME analysis, respectively. A treatment contrast was adopted for all (G)LMEs. The alpha level (α) was 0.05. When the *post-hoc* pairwise comparisons were performed, a Bonferroni correction was used to control the familywise error rate for the multiple comparisons. We report the raw *p*-value and the Bonferroni corrected α when the Bonferroni correction was administered. The *afex* package (Singmann et al., 2021) for R (R Core Team, 2021) was used for (G)LME analyses, and pairwise comparisons were performed using the *emmeans* package (Lenth, 2021). The details of the statistical analysis procedures for each behavioral measure and ERP are described in the following sections.

### 2.6. Subjective rating analysis

Subjective rating data were submitted to a Friedman test to determine whether the levels of goal scores modulated participants' subjective intrinsic motivation and whether the moderate goal was the most motivating—as predicted by SDT theory. When the effect of Goal was significant, pairwise comparisons across levels of goal scores were employed using the Wilcoxon signed-rank test.

### 2.7. Behavioral performance analysis

The behavioral data analyses aimed to reveal whether participants' motivation levels, predicted to be modulated by the levels of the goal scores, affected their behavioral performance. First, we tested whether the scores earned in the sessions differed across levels of goal scores (analysis of Scores). An LME was employed. The response variable was the mean score across the first and second attempts in each level of goal score. Since EEG analysis focused on the data from the two attempts for each level of goal score, analyzing them together in terms of signal-to-noise ratio (SNR) of the ERPs (see the ERP analysis section for more detail), the data from the two attempts were analyzed in combination in all analyses of the behavioral data. Mean scores were log-transformed because of the non-normal distribution of the data. Both Goal and Rating were included as fixed effects.

Second, the frequency of negative feedback (i.e., the number of incorrect or timed-out trials) was submitted to the statistical analysis (analysis of Negative feedback frequency) to determine whether the motivation levels affected the frequency of incorrect answers. The frequency of negative feedback did not follow a normal distribution. Thus, a GLME with the Poisson distribution and the log link function was used. The total number of negative feedback trials between the first and second attempts for a level of goal score was used as the response variable for the analysis. The fixed effects were the same as the analysis of the Scores. In both analyses, if the effect of Goal was significant, pairwise *t*-tests using the Kenward-Roger method in the LME analysis and pairwise *z*-tests in the GLME analysis were performed across possible pairs of goals based on the estimated coefficients.

### 2.8. Analysis of behavioral factors potentially influencing ERPs

Further analyses of behavioral performances were carried out to eliminate the possibility of any confounding differences in performance that would affect feedback-related ERP responses. First, we acknowledged that the expected reward magnitude might affect the FRN (Bellebaum et al., 2010) and P300 (Yeung and Sanfey, 2004). The differences in points earned for each correct answer (i.e., reward magnitude) varied by the number of consecutive correct answers and might have affected our results across the level of goal score. We examined whether the mean points earned for the correct answers in the session differed by the level of goal scores or covariates with subjective ratings (analysis of Reward magnitude). For each level of goal score, the mean points earned for the correct answers within a session were further averaged between the first and second attempts and used as response variables. The values were log-transformed because of the non-normal distribution and submitted to the LME. The fixed effects were Goal and Rating. If the effect of Goal was significant, pairwise *t*-tests using the Kenward-Roger method were performed across possible pairs of goals based on the estimated coefficients.

Second, the temporal local probability of success, calculated based on the probability of success up to a certain trial, affects FRN (Yokota and Naruse, 2021), and P300 amplitudes are also affected by the probability of the target stimuli (Duncan-Johnson and Donchin, 1977). Thus, we examined whether the mean number of consecutive correct answers before the incorrect answer trials in a session (i.e., local frequency of the negative feedback) differed across the level of goal scores or covariates with subjective ratings (analysis of Local outcome frequency). The mean number of consecutive correct answers before incorrect trials within a session was further averaged across the first and second attempts for each level of goal score. The timed-out trials were not considered because they were not used in the EEG analysis (see the ERP analysis section). The non-normal distribution led to log-transformation of values submitted to the LME. The fixed effects were Goal and Rating. If the effect of Goal was significant, pairwise *t*-tests using the Kenward-Roger method were performed across possible pairs of goals based on the estimated coefficients.

Third, we tested for differences in the rate of question type (i.e., the addition of three-digit number pairs, three-digit and two-digit number pairs, and two-digit number pairs) included in the incorrect trials (analysis of Effort magnitude). Previous research has demonstrated that the mental effort for calculation affects the feedback-related ERPs (Ma et al., 2014b; Wang et al., 2017). For this purpose, a GLME was constructed using the Poisson distribution and the log link function. The response variable was the total number of incorrect trials across the first and second attempts per calculation type (Effort: categorical variable). The fixed effects were Goal, Rating, Effort, and interactions of Goal × Effort and Rating × Effort. In the analysis of Effort magnitude, if the effects of Effort were significant, pairwise *z*-tests were performed across possible pairs of the effort levels based on the estimated coefficients. If the effects of Goal were significant, pairwise *z*-tests were performed across possible pairs of the goals. Where significant interactions of Goal × Effort were noted, the pairwise *z*-tests across levels of goal scores were employed for each effort level. If the interaction of Rating × Effort was significant, the significance of Rating coefficients was tested for each effort level.

### 2.9. ERP analysis

EEG data were preprocessed for every session and participant. MATLAB and the FieldTrip toolbox (Oostenveld et al., 2011) were employed for EEG data analysis. The raw EEG and EOG data were high- (filter coefficients = 908, -6 dB cutoff frequency = 1 Hz) and low-pass filtered (filter coefficients = 244, -6 dB cutoff frequency = 30 Hz) using one-pass zero phase Kaiser window sinc finite impulse response filters. Ocular artifacts were corrected using independent component analysis. The filtered data were decomposed into independent components, and the components of ocular artifacts were removed from the data. Artifacts derived from eye movements and blinks were determined by visual inspection of each component waveform and Pearson correlation between each component and EOGs. The EEG data were extracted from -50 to 400 ms relative to the onset of each feedback sound and baseline-corrected using the mean amplitudes of the pre-stimulus time window. Data from two participants in one session were excluded from the analysis because of mechanical trouble with the trigger. Trials with trigger intervals shorter than the epoch length were excluded from the analysis because the baseline region of subsequent trials could be contaminated with ERPs. It is also unlikely that the participants performed the calculations properly in such limited time. The median value of the number of trials removed because of the short epoch length was 2 (first quartile = 0, third quartile = 4). Trials including data points exceeding the range of ±65 μv were excluded from the analysis for artifact rejection. One participant's data were excluded from further ERP analysis because more than 30% of their trials were rejected by this procedure. The median value of the number of rejected trials among the remaining participants was 1 (first quartile = 0, third quartile = 6). The Wilcoxon signed-rank tests showed no significant differences in the number of rejected trials for all pairs of levels of goal score (moderate–hard: *W* = 126, *p* = 0.727, moderate–easy: *W* = 86, *p* = 0.311, hard–easy: *W* = 121, *p* = 0.168, Bonferroni-corrected α = 0.017).

We averaged each participant's trials by the level of goal score and the type of feedback to obtain individuals' ERPs. Trial data were concatenated across two attempts for each level of goal score to ensure a sufficient number of incorrect trials to calculate the ERPs. The timed-out trials were not included in the analysis because it could be predicted in advance that negative feedback would be returned, leading to differences in the cognitive processing of feedback. The mean amplitudes for each ERP were calculated for each participant and the level of goal score. The Fz electrode was used to calculate the mean amplitude of N1 and FRN (fronto-central distribution), and Pz was used to calculate that of P300 (parietal distribution). A small number of trials for calculating the mean amplitude for each level of goal score results in a poor SNR and unreliable results. Thus, if the number of incorrect trials used for ERP calculation was less than 12 for a certain goal score level, that level was excluded from the analysis. If all levels were excluded, the participant was not used in the ERP analysis. The time windows for calculating the mean amplitude of ERPs were defined as from 92 to 132 ms (40 ms length), from 160 to 200 ms (40 ms length), and from 262 to 342 ms (80 ms length) for N1, FRN, and P300, respectively. The center time-point of each time window was the peak latency of each component of the grand average difference ERP waveform. The grand average difference ERP waveform was calculated using trials in all conditions (Luck and Gaspelin, 2017) based on the grand average ERP of negative feedback minus the grand average ERP of positive feedback. The length of the time windows was determined by visual inspection of the grand average difference wave. The minimum number of trials across the levels was randomly selected from each level. The mean amplitude of each level was calculated with these trials to ensure that the SNR of ERPs across conditions did not differ because of differences in the number of trials across levels of goal scores.

The mean amplitudes of N1, FRN, and P300 were submitted to each LME to determine whether differences in participants' motivation levels modified by the level of goal score were reflected in their ERP responses. The fixed effects were Goal, Rating, and Feedback (types of feedback: correct or incorrect), and interactions of Goal × Feedback and Rating × Feedback. The inclusion of Feedback as a fixed effect allowed us to determine whether participants' motivation levels were reflected in the positive or negative feedback ERPs. If the effect of the Goal was significant, pairwise *t*-tests using the Kenward-Roger method were performed across levels of goal scores based on the estimated coefficients. Where a significant interaction of Goal × Feedback was found, pairwise *t*-tests using the Kenward-Roger method across the level of goal scores were conducted for each feedback type. If the interaction of Rating × Feedback was significant, the significance of the estimated coefficient of Rating was determined for each feedback type.

## 3. Results

### 3.1. Subjective ratings

**Figure 3:**
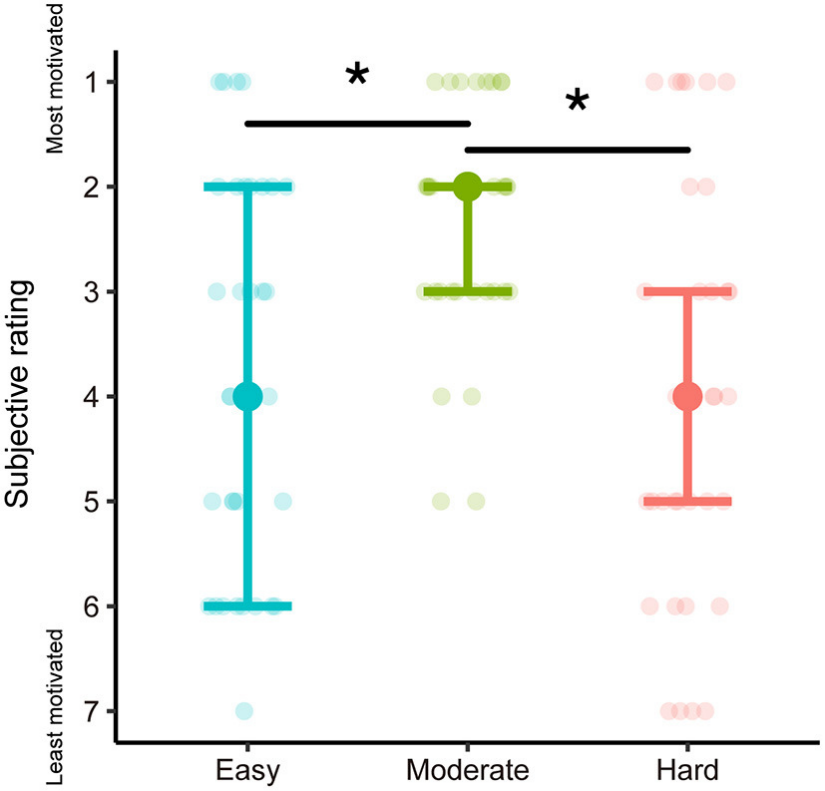
Results of subjective ratings for each level of goal score. Filled dots and error bars indicate median and interquartile range, respectively. The transparent dots are the values for each participant. ^*^p < Bonferroni-corrected α.

The subjective ratings for goal score levels are summarized in Figure 3. The Friedman test revealed a significant effect of Goal [χ^2^_(2)_ = 14.31, *p* = 7.80E-4]. The pairwise Wilcoxon signed-rank test for all pairs of levels of goal score indicated subjective ratings of the moderate goal were significantly higher than those for the easy and hard ones (moderate–easy: *W* = 58, *p* = 8.46E-4, moderate–hard: *W* = 62.5, *p* = 7.53E-4, Bonferroni-corrected α = 0.017). There was no significant difference between easy and hard goals (*W* = 184, *p* = 0.672, Bonferroni-corrected α = 0.017).

The variance in subjective ratings for easy and hard goals was larger than for moderate ones, confirming individual differences in the level of goal score preferences. Therefore, to determine whether this difference in variance was statistically significant, we tested the homogeneity of the variance of subjective ratings for easy and moderate goals and hard and moderate goals, using a Brown-Forsythe test. The Brown-Forsythe test is a modification of the Levene test to increase robustness for non-normality. The variances of the subjective ratings in the easy and hard goals were significantly greater than for the moderate goal [easy–moderate: *F*_(1, 64)_ = 16.22, *p* = 1.52E-4; hard–moderate: *F*_(1, 64)_ = 12.72, *p* = 6.91E-4, Bonferroni-corrected α = 0.025].

The difference in the variance of subjective ratings may be derived from the difference between goals and earned scores. For example, if a participant scores close to the goal score on a hard goal assumed to be hard to achieve, the subjective rating may be relatively higher. To eliminate this possibility, we used LME analysis to determine whether the absolute difference between the goal score and the earned score was related to subjective ratings for the easy and hard goals. The response variable was the absolute value of the difference between the goal and earned scores, log-transformed to approximate a normal distribution. Fixed effects were Attempt (1st attempt and 2nd attempt), Goal, and Rating. The interaction of Attempt × Goal and Attempt × Rating were also included in the model. The LME revealed no significant effects of Rating [*F*_(1, 28.55)_ = 2.09, *p* = 0.159] and no interaction of Attempt × Rating [*F*_(1, 76.94)_ = 1.75, *p* = 0.190]. The effect of Goal was significant [*F*_(1, 101.8)_ = 28.34, *p* = 6.10E-7]—the log-transformed, absolute differences between the goal scores and earned scores were larger for the hard goal than the easy one. The interaction of Goal × Attempt was not significant [*F*_(1, 76.94)_ = 2.28, *p* = 0.135], and the effect of Attempt was marginally significant [*F*_(1, 76.94)_ = 3.49, *p* = 0.066].

### 3.2. Behavioral performance

**Figure 4:**
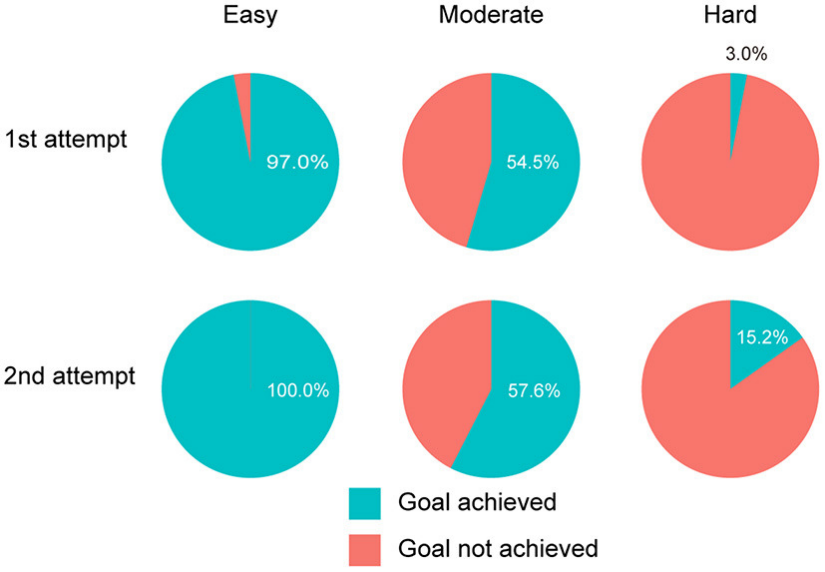
Percentage of participants who achieved the goal score for each level of goal score and attempt. Almost all participants achieved the easy goal and did not achieve the hard one. The percentage of participants who achieved the moderate goal was approximately 50% in both attempts.

The mean values of the goal scores across participants were 40.94 (SD = 27.03), 212.8 (SD = 140.4), and 786.7 (SD = 473.7) for easy, moderate, and hard, respectively. Figure 4 shows the percentage of participants who achieved their goal score for each attempt and the level of goal score. While almost all of them achieved the easy goal, most participants failed to achieve the hard one, and approximately half of the participants reached the moderate goal in each attempt.

**Figure 5:**
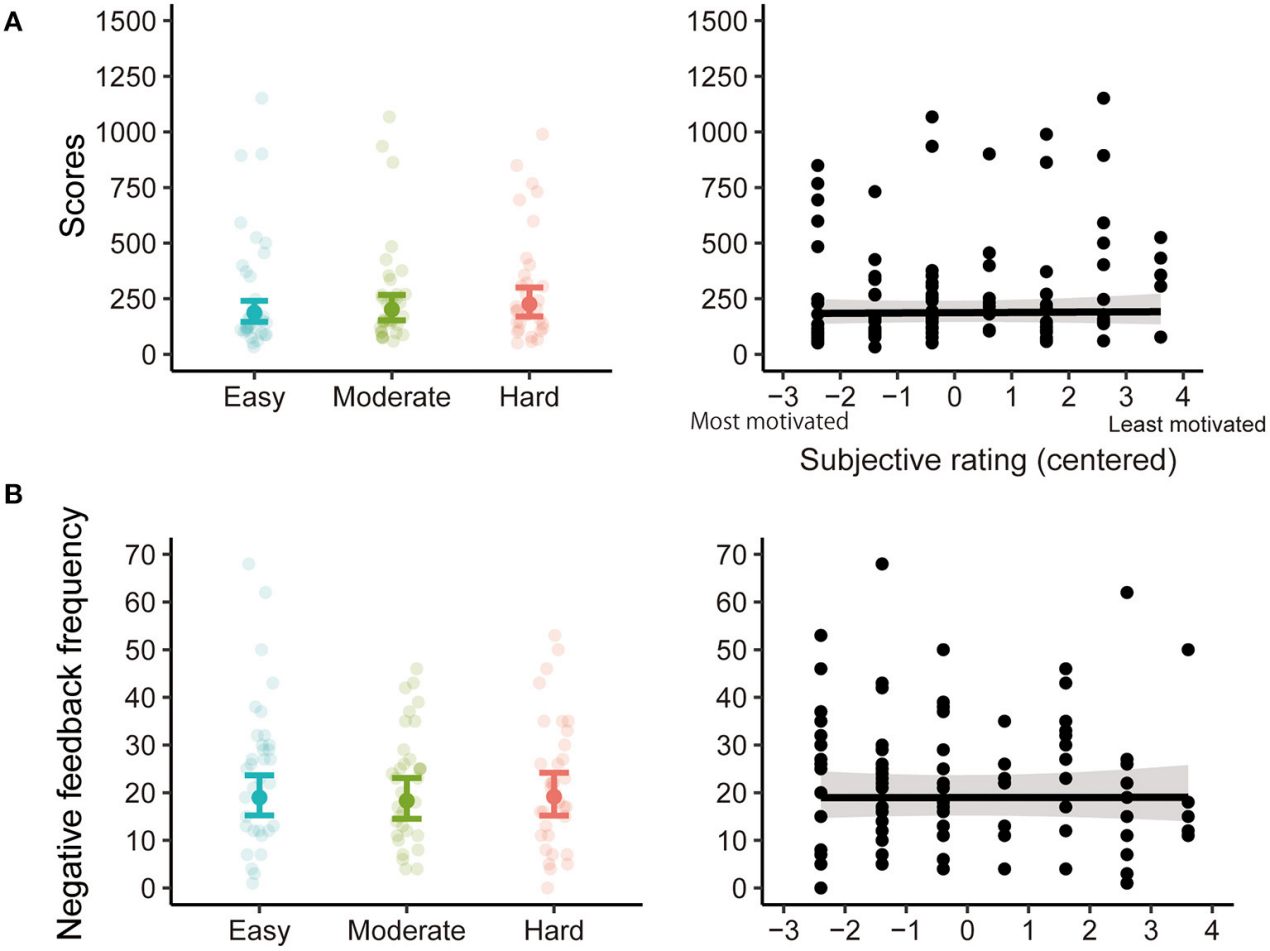
The estimated coefficients of Goal (left) and the predicted values on Rating (right) in the (generalized) linear mixed-effects models [(G)LME] analyses for Scores **(A)** and Negative feedback frequency **(B)**. In Goal results, the filled dots and error bars represent the estimated coefficients and 95% confidence intervals. The transparent dots are data points for each participant. In Rating results, the black line represents the predicted values from the (G)LME, and the gray area represents the 95% confidence interval. Black dots are data points for each participant (number of participants × number of levels of goal scores).

The statistical results of the (G)LME analyses of behavioral performance are summarized in Table 1. Figure 5 depicts the estimated coefficients of Goal (left) and predicted values on Rating (right) in the (G)LMEs. The LME analysis of Scores did not show any significant effect of Goal [*F*_(2, 57.74)_ = 1.37, *p* = 0.263; Figure 5A left] and Rating [*F*_(1, 31.10)_ = 0.035, *p* = 0.854; Figure 5A right]. In the GLME analysis of Negative feedback frequency, both effects of Goal [χ^2^_(2)_ = 0.521, *p* = 0.771; Figure 5B left] and Rating [χ^2^_(1)_ = 0.001, *p* = 0.982; Figure 5B right] were also not significant.

**Table 1:** Summary of statistical analyses for behavioral performances.

	**Test**	**Fixed effects**	**Statistic**	**d.f**.	***p***
Scores	*F*-test	Goal	1.37	(2, 57.74)	0.263
		Rating	0.035	(1, 31.10)	0.854
Negative feedback frequency	LRT	Goal	0.521	2	0.771
		Rating	0.001	1	0.982

d.f., the degrees of freedom; LRT, the likelihood ratio test.

The statistics of the F-test and LRT are F and χ^2^, respectively.

### 3.3. Behavioral factors potentially influencing ERP

**Figure 6:**
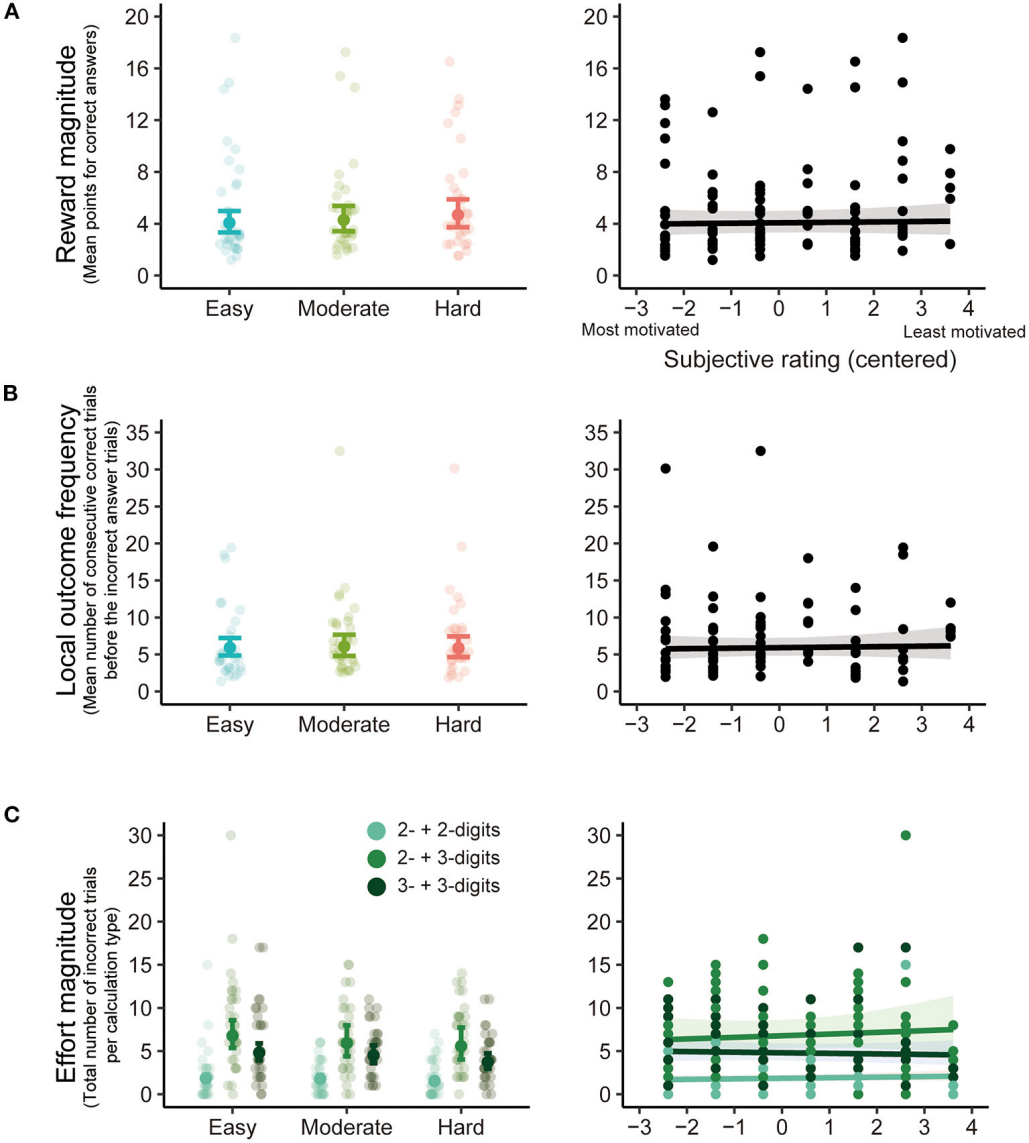
The estimated coefficients of Goal (left) and the predicted values on Rating (right) in the (generalized) linear mixed-effects models [(G)LME] analyses for Reward magnitude **(A)**, Local outcome frequency **(B)**, and Effort magnitude **(C)**. In Goal results, the filled dots and error bars represent the estimated coefficients and 95% confidence intervals. The transparent dots are data points for each participant. In Rating results, the black line represents the predicted values from the (G)LME, and the gray area represents the 95% confidence interval. Black dots are data points for each participant (number of participants × number of levels of goal scores).

The statistical results of the (G)LME analyses on the behavioral factors potentially influencing ERP are summarized in Table 2. Figure 6 depicts the estimated coefficients of Goal (left) and predicted values on Rating (right) in the (G)LMEs. The results of Reward magnitude showed no significant effects of Goal [*F*_(2, 56.78)_ = 1.17, *p* = 0.317; Figure 6A left] and Rating [*F*_(1, 31.39)_ = 0.081, *p* = 0.778; Figure 6A right]. Local outcome frequency also showed no significant fixed effects [Goal: *F*_(2, 52.85)_ = 0.044, *p* = 0.958, Figure 6B left; Rating: *F*_(1, 32.06)_ = 0.079, *p* = 0.780, Figure 6B right].

**Table 2:** Summary of statistical analyses of behavioral factors potentially influencing event-related potentials.

	**Test**	**Fixed effects**	**Statistic**	**d.f**.	***p***
Reward magnitude	*F*-test	Goal	1.17	(2, 56.78)	0.317
		Rating	0.081	(1, 31.39)	0.778
Local outcome frequency	*F*-test	Goal	0.044	(2, 52.85)	0.958
		Rating	0.079	(1, 32.06)	0.780
Effort magnitude	LRT	Goal	5.70	2	0.058
		Rating	0.289	1	0.591
		Effort	297.0	2	3.24E-65^*^
		Goal × Effort	0.710	4	0.950
		Rating × Effort	1.53	2	0.465

d.f., the degrees of freedom; LRT, the likelihood ratio test.

* p < 0.05.

The statistics of the F-test and LRT are F and χ^2^, respectively.

Effort magnitude reports indicated that the effect of Effort was, unsurprisingly, significant (χ^2^(2) = 297.0, *p* = 3.24E-65; Figure 6C left). Pairwise *z*-tests across effort levels showed that incorrect addition of the two- and three-digit number pairs was significantly more frequent than for two-digit number pairs (two- and three-digit number pairs—two-digit number pairs: *z* = 15.70, *p* = 1.59E-55, Bonferroni-corrected α = 0.017) and the three-digit number pairs (two- and three-digit pairs—three-digit number pairs: *z* = 5.79, *p* = 6.95E-9, Bonferroni-corrected α = 0.017) because there were more two- and three-digit numbers than other pairs in the task (c.f. Figure 1). The number of incorrect trials for three-digit number pairs was significantly higher than for two-digit number pairs (three-digit number pairs—two-digit number pairs: *z* = 10.98, *p* = 4.65E-28, Bonferroni-corrected α = 0.017), possibly because three-digit number pairs are more difficult to calculate than two-digit number pairs. The effect of Goal approximated significance (χ^2^(2) = 5.70, *p* = 0.058) while other fixed effects did not reach significance (all other χ^2^s ≤ 1.53; all other *p*s ≥ 0.465).

### 3.4. ERPs

**Figure 7:**
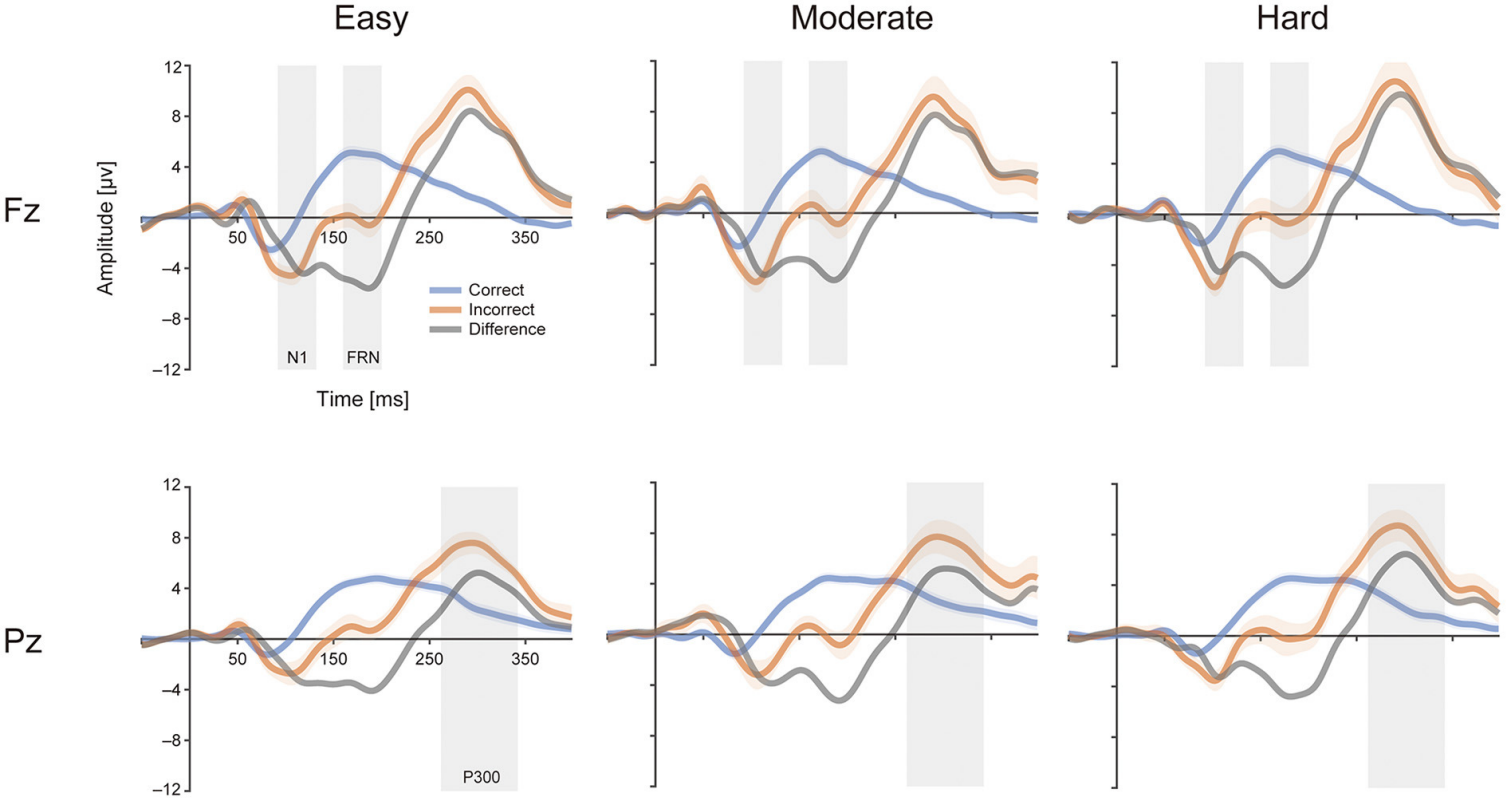
The grand average event-related potentials (ERPs) for correct and incorrect trials time-locked to the feedback onset and their difference waveforms for each channel and level of goal score. The blue and red area represents the standard error for correct and incorrect trials, and the gray rectangles represent the range of the time window for calculating the mean amplitude of each ERP. For each level of goal score, participants with fewer than 12 incorrect trials were excluded from the calculation of ERPs for incorrect trials at that level of goal score in terms of signal-to-noise ratio.

**Figure 8:**
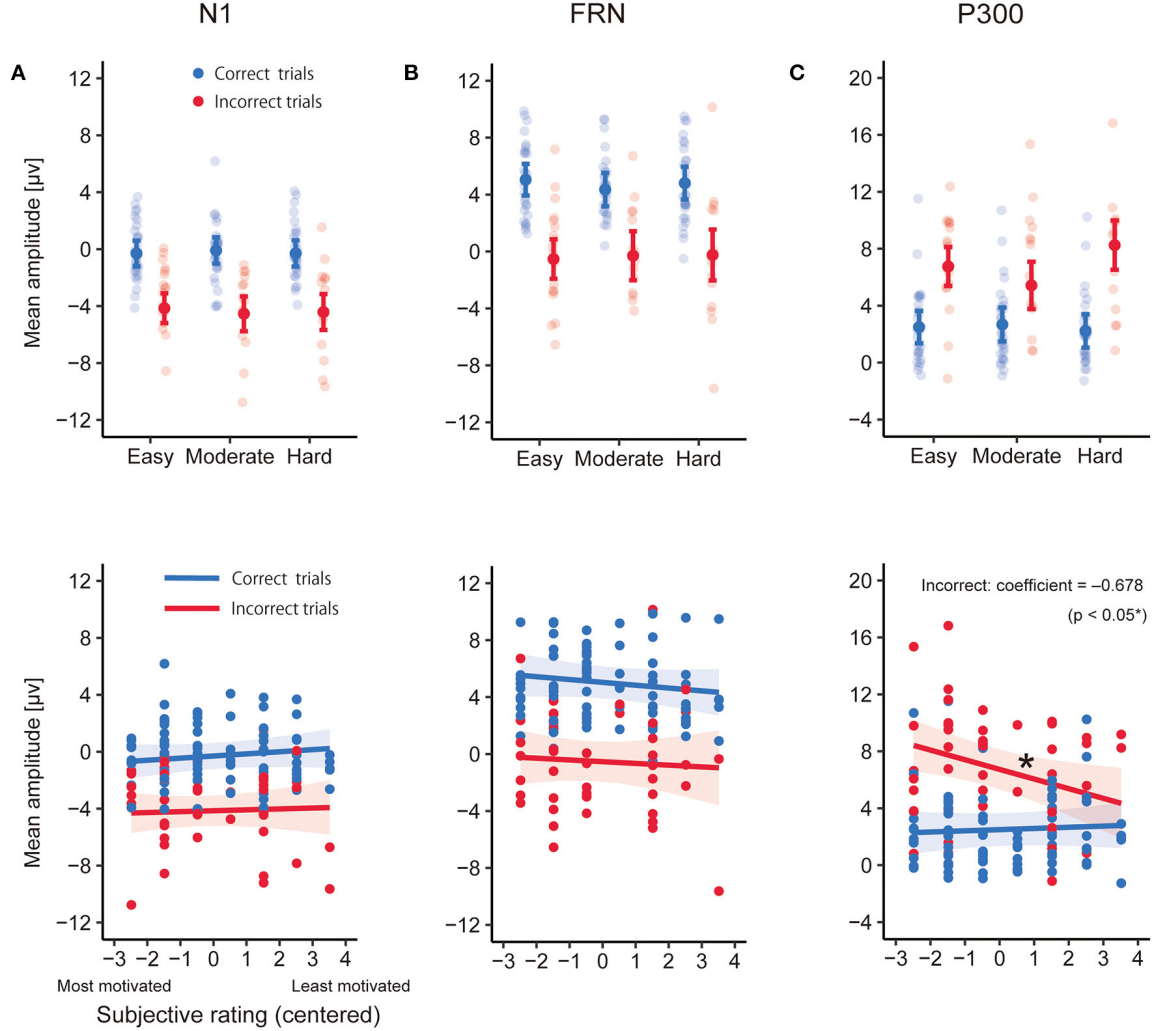
The estimated coefficients of Goal (upper) and the predicted values on Rating (lower) for each incorrect and correct trial in the linear mixed-effects models (LME) analyses for the mean amplitudes of N1 **(A)**, feedback-related negativity (FRN) **(B)**, and P300 **(C)**. In Goal results, the filled dots and error bars represent the estimated coefficients and 95% confidence intervals. The transparent dots are data points for each participant. In Rating results, the colored line represents the predicted values from the LME, and the colored area represents the 95% confidence interval. Filled dots are data points for each participant (number of participants × number of levels of goal scores for each feedback type). ^*^p < 0.05.

Figure 7 shows the grand average ERPs for correct and incorrect trials time-locked to feedback onset. Their difference waveforms are also shown for each channel and level of goal score. The results of the statistical analyses are summarized in Table 3. Figure 8 shows the estimated coefficients of Goal (upper) and predicted values on Rating (lower) in the LMEs for each mean amplitude. The LME analysis for N1 components showed a significant effect of Feedback [*F*_(1, 83.85)_ = 165.9, *p* = 1.42E-21]. The N1 amplitude of the negative feedback showed more negative deflection than the positive feedback. No other significant effects were found (all other *F*s ≤ 0.482; all other *p*s ≥ 0.492) (Figure 8A).

**Table 3:** Summary of statistical analyses (*F*-tests) of the mean amplitudes of event-related potentials.

		**Fixed effects**	***F***	**d.f**.	***p***
N1		Goal	0.051	(2, 98.67)	0.950
		Rating	0.482	(1, 40.62)	0.492
		Feedback	165.9	(1, 83.85)	1.42E-21^*^
		Goal × Feedback	0.314	(2, 81.22)	0.731
		Rating × Feedback	0.157	(1, 85.99)	0.693
FRN		Goal	0.077	(2, 105.6)	0.926
		Rating	0.748	(1, 122.4)	0.389
		Feedback	98.25	(1, 110.8)	5.79E-17^*^
		Goal × Feedback	0.292	(2, 102.7)	0.747
		Rating × Feedback	0.052	(1, 106.3)	0.821
P300		Goal	1.36	(2, 102.4)	0.263
		Rating	2.95	(1, 115.0)	0.088
		Feedback	83.88	(1, 106.0)	4.38E-15^*^
		Goal × Feedback	2.74	(2, 100.4)	0.069
		Rating × Feedback	5.93	(1, 102.8)	0.017^*^

d.f., the degrees of freedom.

* p < 0.05.

The FRN analysis confirmed the significant effect of Feedback [*F*_(1, 110.8)_ = 98.25, *p* = 5.79E-17], where the mean FRN amplitudes of the negative feedback showed significantly more negative deflection than for positive feedback. No other significant effects were found (all other *F*s ≤ 0.748; all other *p*s ≥ 0.389) (Figure 8B).

Analysis of P300 showed a significant effect of Feedback [*F*_(1, 106.0)_ = 83.88, *p* = 4.38E-15], with more positive deflection for negative feedback than for positive. The effect of Rating was marginally significant [*F*_(1, 115.0)_ = 2.95, *p* = 0.088], and the effect of Goal was not significant [*F*_(2, 102.4)_ = 1.36, *p* = 0.263]. The effect of Goal × Feedback approximated significance [*F*_(2, 100.4)_ = 2.74, *p* = 0.069]. There was a significant interaction of Rating × Feedback [*F*_(1, 102.8)_ = 5.93, *p* = 0.017]. The coefficients of Rating reached significance for the negative feedback [β = -0.678, *t*_(109)_ = -2.43, *p* = 0.017]. The coefficients, however, did not demonstrate significance for positive feedback [β = 0.083, *t*_(111)_ = 0.470, *p* = 0.639; Figure 8C]. The estimated coefficients and 95% intervals in LMEs are summarized in Table 4.

**Table 4:** Estimated coefficients (β) and the 95% confidence interval in the linear mixed-effects models of the mean amplitudes of event-related potentials.

	**N1**		**FRN**		**P300**	
**Predictors**	**β**	**CI**	**β**	**CI**	**β**	**CI**
FB[cor.] (Intercept)	-0.300	[-1.20, 0.599]	5.04	[3.93, 6.15]	2.50	[1.36, 3.64]
FB[incor.]	-3.85	[-4.84, -2.85]	-5.57	[-7.18, -3.97]	4.23	[2.77, 5.70]
Rating	0.149	[-0.147, 0.446]	-0.199	[-0.569, 0.170]	0.083	[-0.260, 0.426]
Goal[mod.]	0.211	[-0.707, 1.13]	-0.694	[-2.15, 0.761]	0.181	[-1.14, 1.50]
Goal[hard]	0.006	[-0.911, 0.923]	-0.236	[-1.63, 1.15]	-0.270	[-1.53, 0.990]
FB[incor.]: Rating	-0.086	[-0.506, 0.335]	0.078	[-0.590, 0.745]	-0.761	[-1.37, -0.150]
FB[incor.]: Goal[mod.]	-0.603	[-2.11, 0.901]	0.924	[-1.53, 3.38]	-1.51	[-3.74, 0.720]
FB[incor.]: Goal[hard]	-0.274	[-1.86, 1.31]	0.526	[-2.04, 3.09]	1.77	[-0.571, 4.11]

CI, 95% confidence interval; FB, Feedback; cor., correct; incor., incorrect; mod., moderate.

## 4. Discussion

This study examined whether ERP measures are modulated by the challenge level of the goal scores determined by participants' competence or are related to the preference for these goal levels when using a gamified educational application. The achievement percentages for each level of goal score showed that almost all participants achieved the goal score for the easy goal, while almost no one reached the hard goal. In contrast, approximately half the participants achieved the moderate goal, indicating that the moderate goal was not biased toward either difficult or easy. Participants also reported that they were most motivated to achieve moderate goals, as predicted by the SDT and supporting the validity of the goal scores in the current research. The ERP results indicated that the P300 amplitude in response to negative feedback is significantly related to subjective ratings of achievement motivation for each level of goal score rather than the level of goal scores themselves.

P300 amplitude reflects the motivational significance of the target stimuli (Nieuwenhuis et al., 2005) and is also enhanced by increased allocation of attentional resources to target stimuli (Nieuwenhuis et al., 2005; Polich, 2007). Thus, it is likely that as participants' intrinsic motivation to achieve the goal scores increased, their allocation of attentional resources to negative feedback increased and/or negative feedback was perceived as more motivationally significant information for goal achievement.

These motivation effects on the P300 amplitudes are consistent with previous research (Kleih et al., 2010; Fang et al., 2020). The results of the subjective ratings showed that moderate goals were the most motivating compared with easy and hard goals. Despite the subjective rating results, modulation of ERP amplitude by the degree of intrinsic motivation was observed as a covariate with subjective ratings rather than the levels of the goal scores. The variance in subjective ratings was significantly greater for easy and hard goals than for moderate goals. This difference in variance was not related to the proximity of the earned score to the goal score. Thus, even though most participants were willing to achieve moderate goals, the degree of motivation for easy and hard goals may depend on participants' preferences. One group may prefer a level of goal score that is difficult to achieve, while another may prefer a level below their competence. The P300 indicators successfully captured such preferences for the level of goal scores in the gamified educational application.

The current research did not find any relationship between FRN and intrinsic motivation linked to the levels of goal scores—in contrast with P300. Positive emotions increase FRN responses because a positive mood decreases the expectation of negative feedback (Paul and Pourtois, 2017). Boredom states also affect the response (Milyavskaya et al., 2019). Thus, the finding that FRN was not affected by the levels of goal score suggests, at least, that the settings of the levels of goal scores did not affect participants' boredom and positive emotions during the current task. However, previous studies have reported FRN's role as indicating intrinsic motivation (Ma et al., 2014a; Fang et al., 2018; Meng et al., 2021) and have yielded inconsistent results regarding whether changes in the degree of intrinsic motivation are reflected in P300. Contrary to the present results, several studies failed to observe the effect of the degree of intrinsic motivation on P300 amplitude. For example, Ma et al. (2014a) reported that the reduction in intrinsic motivation, brought about by the undermining effect of monetary rewards, is reflected in the FRN, not the P300 amplitude to outcome feedback. Fang et al. (2021) also showed that a change in intrinsic motivation due to negative information about participants' task performance based on comparison with others—such as “your performance was below average”—did not affect the P300 amplitude time-locked to outcome feedback. The discrepancy between these previous studies and the current result may be because of the method used to change the participants' intrinsic motivation. In this study, participants were presented with a goal score prior to the session and encouraged to exceed that score. Such instructions might have increased the allocation of attentional resources to negative feedback when they were more intrinsically motivated to achieve goals, because negative feedback for each answer was motivationally significant information for whether the goal could be achieved. Related to this result, Leng and Zhou (2010) reported the impact of interpersonal relationships on outcome evaluation during a gambling task, i.e., whether observing a friend or stranger's outcome feedback was reflected in the P300 only and not in FRN. Findings from that study suggested that outcome evaluation can be divided into an early semi-automatic process reflected in FRN and a later outcome evaluation process sensitive to the allocation of attentional resources, reflected in P300 (Leng and Zhou, 2010). As in their result, changes in intrinsic motivation due to levels of goal scores may affect only the later cognitive processes reflected in the P300, not the early semi-automatic processes in outcome evaluation. It should be noted, however, that the failure to observe the effect of the intrinsic motivation to achieve the goal scores on FRN may be due to the number of trials used in the analysis. In the present analysis, we used at least 12 trials to calculate ERP waveforms for each level of goal scores. This is a relatively small number compared with typical ERP studies, and to observe reliable FRN responses, 20 trials are desirable (Marco-Pallares et al., 2010). The lack of FRN effects may be because of the small number of trials, given the amplitude of the FRNs was generally smaller than P300. Taken together, the current result suggests that the measurement of P300 amplitude is most appropriate for using neural indicators to set the appropriate level of goal scores to foster users' intrinsic motivation in educational gamification applications. The usefulness of FRNs for this purpose will be addressed in future studies.

Behavioral performance—earned scores and negative feedback frequency—did not differ significantly between the levels of goal scores and was not related to subjective ratings. Thus, at least for the task in this study, differences in intrinsic motivation did not affect behavioral performance. These behavioral results suggest that even though motivation levels varied by the levels of the goals, participants were engaged in the task even if the goals were easier or harder to achieve. When considering a system that adaptively sets goal scores for each learner based on their degree of intrinsic motivation, ERP measurement is superior to measurements of behavioral performance. One might argue that the P300 results for the negative feedback were derived from differences in the behavioral performances affecting the feedback-related ERPs. However, analyses of the possible factors influencing these ERPs, such as reward magnitude, local outcome frequency, and effort magnitude, showed no significant relationship with subjective ratings, ruling out the possibility that the P300 results obtained were confounded by these behavioral factors.

The P300 effect was observed only for negative feedback. However, Fang et al. (2020) showed that P300 motivational effects were noted regardless of outcome valence, and other studies indicate that P300 is sensitive to the magnitude of the reward, not the outcome valence (Yeung and Sanfey, 2004; Sato et al., 2005). However, the sensitivity of P300 to outcome valence remains controversial and may be influenced by outcome probability (San Martín, 2012). In Fang et al. (2020)'s research, the probability of success in a task was approximately 50%. However, in the current study, the number of incorrect trials was lower than the number of correct trials. In addition, our gamified GUI application was designed to return sequential feedback immediately after the answer to a calculation was entered. This sequential feedback was similar to the oddball task, where P300 was observed for infrequent target stimuli. Thus, the frequency of negative feedback and the sequential presentation of feedback—like in the oddball task—might produce P300 in response to negative feedback.

A limitation of this study is that we did not consider anxiety levels or history of psychiatric disorders in the study participants. Participants were healthy and had no history of neurological disease, but further information about their mental health was not gathered. Studies have shown that individuals with depression (Keren et al., 2018)—even remitted depression (Santesso et al., 2008)—and high trait anxiety (Gu et al., 2010; Takács et al., 2015) modulate the FRN. These factors might have influenced the feedback-related ERPs observed in the current study, and will be taken into account in future studies.

In conclusion, this study showed that EEG measurements during a gamified educational GUI application could reflect the modulation of intrinsic motivation by the levels of the goal scores. P300 amplitudes varied based on a preference for each level of goal score rather than the levels of goal scores. The results indicate individual differences in preference for the level of goal scores and that P300 can serve as an indicator to capture these individual differences. Using this neural indicator, it may be possible to adaptively set each learner's goal score in gamified educational applications to reinforce learners' intrinsic motivation. Setting goal scores in line with individual preferences may help improve and maintain learners' motivation when engaged in gamification educational applications.

## Data availability statement

The datasets presented in this article are not readily available because part of this data cannot be shared without the participants' consent. Requests to access the datasets should be directed to YN, y_naruse@nict.go.jp.

## Ethics statement

The studies involving human participants were reviewed and approved by the Ethics Committee for Human and Animal Research of the National Institute of Information and Communications Technology. The patients/participants provided their written informed consent to participate in this study.

## Author contributions

HW wrote the manuscript. All authors were involved in the experimental design, data analysis, interpretation, discussion of the results, reviewed, and approved the final version of the manuscript.

## Funding

This work was partially supported by JSPS KAKENHI Grant No. JP20K14110.

## Conflict of interest

The authors declare that the research was conducted in the absence of any commercial or financial relationships that could be construed as a potential conflict of interest.

## Publisher's note

All claims expressed in this article are solely those of the authors and do not necessarily represent those of their affiliated organizations, or those of the publisher, the editors and the reviewers. Any product that may be evaluated in this article, or claim that may be made by its manufacturer, is not guaranteed or endorsed by the publisher.
